# Myeloperoxidase‐Antibody Positivity and Progression to Microscopic Polyangiitis in Interstitial Lung Disease: A Case Series

**DOI:** 10.1002/rcr2.70271

**Published:** 2025-07-09

**Authors:** Michael Hoskins, Grace Thompson, Vidya Navaratnam

**Affiliations:** ^1^ Respiratory Department Sir Charles Gairdner Hospital Perth Australia; ^2^ Institute for Respiratory Health The University of Western Australia Perth Australia; ^3^ Department of Immunology Sir Charles Gairdner Hospital Perth Australia; ^4^ Department of Immunology PathWest, Nedlands Perth Australia

**Keywords:** ANCA associated vasculitis, interstitial lung disease, microscopic polyangiitis, myeloperoxidase, progressive pulmonary fibrosis

## Abstract

Microscopic polyangiitis (MPA) is associated with myeloperoxidase‐antibody positivity and pulmonary complications. However, these antibodies may be found incidentally in some people with interstitial lung disease (ILD). As a result, management of people with ILD and myeloperoxidase‐antibody positivity remains unclear. Our case series describes the natural history of this group of patients over a mean follow‐up period of 4 years. Using data from a public sector pathology provider in Western Australia, we identified 17 people with a positive myeloperoxidase‐antibody titre and who attended our tertiary Respiratory outpatient clinic. Eventual or concurrent diagnosis of MPA was more common in those with radiological usual interstitial pneumonia pattern, higher baseline and peak myeloperoxidase‐antibody titres, lower gas transfer capacity and lower distances achieved on 6‐min walk testing. These features may be used by clinicians during multidisciplinary team discussion to identify those at high‐risk of developing MPA.

## Graphical abstract

Microscopic polyangiitis (MPA) is associated with myeloperoxidase‐antibody positivity and pulmonary complications. Our case series describes the natural history of 17 patients with interstitial lung disease (ILD) and a positive myeloperoxidase‐antibody titre, over a mean follow‐up period of 4 years to help with a better understanding of the management of these patients.

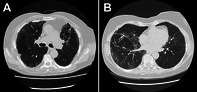

## 1 Introduction

Microscopic polyangiitis (MPA) is a pauci‐immune antineutrophil cytoplasmic antibody (ANCA) associated vasculitis (AAV) predominantly affecting the small vessels [1]. Myeloperoxidase (MPO) antibodies are present in around 60%–80% of those with MPA are associated with a higher risk of development of vasculitis among those with interstitial lung disease (ILD) [2]. People with MPA appear to have more severe systemic disease, with higher rates of pulmonary involvement and mortality [3].

Approximately 42% of those with MPA develop pulmonary complications, most commonly ILD [3, 4]. Usual interstitial pneumonia (UIP) is the most common radiological pattern and is associated with poor prognosis [5, 6, 7]. Pulmonary function testing generally shows a restrictive pattern, with decreased lung volumes and gas transfer capacity which decline with progression over time [4].

Antifibrotic treatment has become part of standard care for those with progressive pulmonary fibrosis, to reduce the rate of lung function decline. It is now recognised that without an autoimmune driver to fibrotic lung disease, immunosuppression should be avoided to reduce unnecessary risk of complications, including infection [8]. MPA treatment sits in stark contrast to this, requiring immunosuppression to prevent further morbidity and mortality from systemic inflammation [9]. Therefore, delineating primary fibrotic from inflammatory‐driven interstitial lung disease has important treatment implications.

Many people with ILD and MPO‐antibody positivity never progress to clinically significant MPA‐ILD, termed isolated ANCA‐ILD. Current guidelines do not suggest routine ANCA testing in newly diagnosed ILD in the absence of features of vasculitis; however, recent rheumatological guidelines suggest ANCA testing in anyone with idiopathic interstitial pneumonia [10, 11]. It is likely that this has led to underdiagnosis of isolated ANCA‐ILD and it remains unclear if these people are at higher risk of later progression to MPA‐ILD or require closer surveillance. This case series aims to describe the clinical practice of this subset of patients in a large tertiary ILD centre and describe the natural history of MPO‐antibody associated ILD.

## 2 Case Series

### 2.1 Methods

A retrospective search of the Western Australian public sector pathology laboratory information system was performed from January 2016 to December 2023 to identify all people who had recorded a positive MPO‐antibody titre (> 3.5 units/mL) and had attended the Sir Charles Gairdner Hospital Respiratory outpatient clinic for a multidisciplinary team (MDT) consensus diagnosis of ILD. MPO‐antibody testing was performed by ELiA MPO fluroenzymeimmunoassay. Medical records were retrospectively reviewed from December 2024, back to the earliest of either ILD diagnosis or MPO‐antibody positivity. Data collected included demographics, clinical features and treatments, radiology, laboratory results and pulmonary function testing. Radiological patterns were defined based upon established guidelines with consensus achieved for each case at an ILD multidisciplinary team meeting [10]. Descriptive analyses were performed, with parametric data presented as mean (±standard deviation) and non‐parametric data as median (interquartile range, IQR). Paired, two‐tailed Student's *t*‐tests were used to assess for significance, with a significance threshold of *p* < 0.05.

### 2.2 Results

Seventeen people were identified for inclusion. Eight (47.1%) received a clinical diagnosis of MPA‐ILD after consensus multidisciplinary Radiology, Respiratory and Immunology discussion based upon the 2012 Chapel Hill consensus criteria [12]. Mean time from ILD diagnosis until time of retrospective review was 4.6 ± 3 years. Those with MPA‐ILD were more likely to be older, ex/current smokers and had similar rates of cardiovascular or airways disease (see Table 1). People with MPA‐ILD also appeared to have a higher baseline erythrocyte sedimentation rate (ESR), although this did not reach statistical significance.

**TABLE 1:** Demographics data and baseline characteristics.

	All patients (*n* = 17)	MPA‐ILD (*n* = 8)	Isolated ANCA‐ILD (*n* = 9)	*p*
Demographics				
Mean age at diagnosis—years (std dev)	70.24 (11.08)	75.88 (9.52)	65.22 (10.31)	
Male—no. (%)	9 (53)	6 (75)	3 (33)	
Current or previous smoker at time of diagnosis—no. (%)	9 (53)	6 (75)	3 (33)	
Mean pack year history—pack years (std dev)	21 (28)	35 (30)	10 (20)	
Mean follow up period—months (std dev)	55.39 (35.63)	62.5 (36.27)	49.07 (35.94)	
Median total hospital admissions during follow up—no. (IQR)	3 (1–5)	3 (3–9)	2 (1–3)	**0.046**
Median hospital admissions for respiratory cause during follow up—no. (IQR)	2 (1–3.75)	2.5 (2–4.5)	1 (1–2.5)	0.130
Comorbidities				
Cardiovascular disease—no. (%)	9 (53)	4 (50)	5 (56)	
Airways disease—no. (%)	5 (29)	3 (37)	2 (22)	
Prior malignancy—no. (%)	6 (35)	3 (37)	3 (33)	
Biochemistry				
Mean ESR at diagnosis—mm/h (std dev)	28.48 (44.91)	38 (20.9)	30.33 (32.75)	0.488
Mean baseline MPO‐Ab titre—U/mL (std dev)	86.78 (156.59)	153.45 (213.77)	27.52 (25.66)	0.099
Mean peak MPO‐Ab titre—U/mL (std dev)	123.46 (185.51)	225.63 (233.29)	29.39 (27.85)	**0.032**
Mean time to peak MPO‐Ab titre from initial diagnosis—months (std dev)	16.59 (22.49)	16.58 (20.1)	16.60 (25.66)	0.987
Perinuclear or mixed ANCA staining at diagnosis—no. (%)	13 (76)	7 (87)	6 (67)	
Antinuclear antibody positivity—no. (%)	7 (41)	4 (50)	3 (33)	
Radiological findings				
Definite UIP radiological pattern at diagnosis—no. (%)	1 (6)	1 (13)	0 (0)	
Probable UIP radiological pattern at diagnosis—no. (%)	4 (24)	3 (37)	1 (11)	
Alternative to UIP pattern at diagnosis—no. (%)	12 (71)	4 (50)	8 (89)	
Baseline respiratory function				
Baseline FEV1—L (std dev)	2.17 (0.7)	2.22 (0.55)	2.12 (0.86)	0.797
FEV1% of predicted—% (std dev)	81.78 (21.58)	87.01 (21.67)	76.55 (21.57)	0.347
Baseline FVC—L (std dev)	2.80 (0.91)	2.94 (0.8)	2.65 (1.05)	0.539
FVC% of predicted—% (std dev)	80.98 (23.56)	86.78 (23.57)	75.18 (23.60)	0.340
Baseline TLC—L (std dev)	4.61 (1.04)	4.79 (0.91)	4.26 (1.33)	0.517
TLC% of predicted—% (std dev)	82.65 (12.26)	80.23 (19.27)	87.5 (21.14)	0.565
Baseline DLCO—mL/min/mmHg (std dev)	15.32 (3.46)	14.3 (3.43)	16.96 (3.14)	0.189
DLCO% of predicted—% (std dev)	68.4 (18.63)	64.02 (20.1)	75.4 (15.32)	0.303
Baseline 6MWT distance—m (std dev)	415 (121.63) (*n* = 9)	365.17 (79.04) (*n* = 6)	510 (172.84) (*n* = 3)	0.116
Treatment				
Immunosuppression use—no. (%)	12 (71)	8 (100)	4 (44)	
Glucocorticoids—no. (%)	10 (59)	6 (75)	4 (44)	
Mycophenolate—no. (%)	3 (18)	2 (25)	1 (11)	
Cyclophosphamide—no. (%)	3 (18)	3 (37)	0 (0)	
Rituximab—no. (%)	5 (29)	5 (62)	0 (0)	
Antifibrotic use (nintedanib)—no. (%)	1 (6)	1 (12)	0 (0)	

*Note:* Bold values meeting significant threshold of *p* < 0.05.

Abbreviations: 6MWT, 6‐min walk test; ANCA, antineutrophil cytoplasmic antibody; DLCO, diffusion capacity for carbon monoxide; ESR, erythrocyte sedimentation rate; FEV1, forced expiratory volume in 1 s; FVC, forced vital capacity; ILD, interstitial lung disease; IQR, interquartile range; MPA, microscopic polyangiitis; MPO‐Ab, myeloperoxidase antibody; NSIP, nonspecific interstitial pneumonia; TLC, total lung capacity; UIP, usual interstitial pneumonia.

**FIGURE 1:**
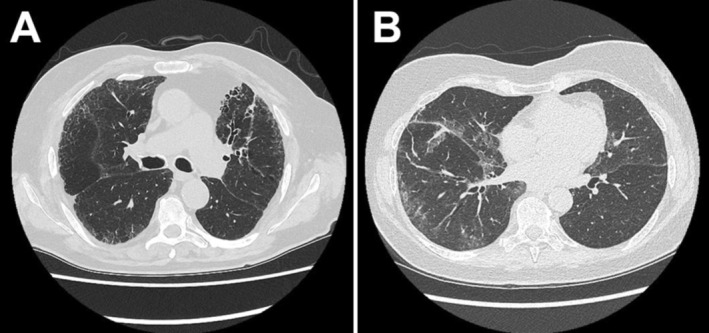
Radiological patterns in ILD. Definite UIP radiological pattern (A) in a person with isolated ANCA‐ILD demonstrating subpleural reticulation, traction bronchiectasis and honeycombing. They later progressed to a diagnosis of MPA‐ILD. Alternate radiological patterns to UIP were more common among those with isolated ANCA‐ILD, with fibrotic non‐specific interstitial pneumonia pattern pictured here (B) demonstrating asymmetrical peribronchovascular ground glass infiltrate with subpleural sparing.

People with MPA‐ILD were more likely to have a UIP radiological pattern at diagnosis (see Figure 1). Only one person who presented with a UIP pattern did not progress to a diagnosis of MPA‐ILD. There was heterogenous radiological patterns among those with isolated ANCA‐ILD, including non‐specific interstitial pneumonia, unclassifiable ILD and organising pneumonia. Of the eight people diagnosed with MPA‐ILD during follow‐up, 62.5% had an established MDT diagnosis of isolated ANCA‐ILD before progression to MPA‐ILD.

**FIGURE 2:**
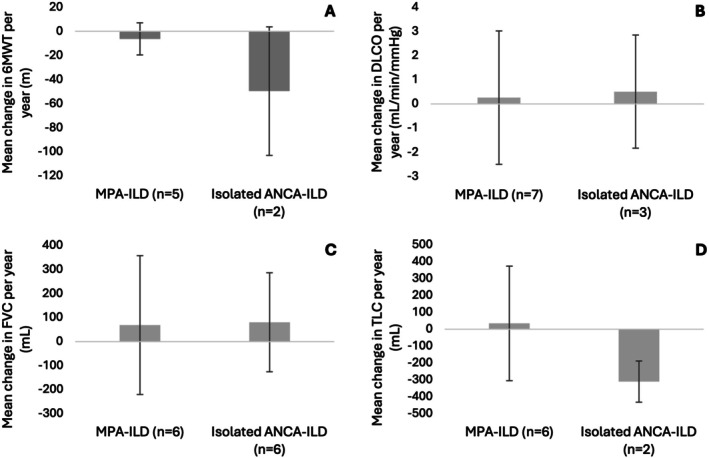
Functional and lung function testing over time in people with MPA‐ILD and isolated ANCA‐ILD. Those with MPA‐ILD had slower decline in 6MWT (−6.3 ± 13.3 vs. −49.6 ± 53.3 m/year) (A). There was similar change in gas transfer capacity (0.3 ± 2.8 vs. 0.5 ± 2.3 mL/min/mmHg/year) (B) and forced vital capacity (69.0 ± 289.2 vs. 80.6 ± 206.2 mL/year) (C). Those with MPA‐ILD appeared to maintain relatively stable total lung capacity over time compared to a slow decline in those with isolated ANCA‐ILD (34.3 ± 339.3 vs. −310.4 ± 122.1 mL/year) (D). Error bars represent standard deviation.

Our cohort had a median of three pulmonary function tests performed (IQR 2–4) during follow up. MPA‐ILD patients tended to have lower diffusion capacity for carbon monoxide (DLCO) and lower distance achieved on the 6‐min walk test (6MWT) at baseline. Compared with MPA‐ILD, those with isolated ANCA‐ILD had a greater loss in lung volume, as measured by total lung capacity, and greater reduction in 6MWT distance per year (see Figure 2).

All people with MPA‐ILD were immunosuppressed, with glucocorticoids given in 75%, rituximab in 62% and infrequent use of cyclophosphamide and mycophenolate. 44% of those with isolated ANCA‐ILD were immunosuppressed. Of these, all received glucocorticoids and 11% received mycophenolate in addition. Antifibrotics were used in one (12%) of those with MPA and not prescribed to any with isolated ANCA‐ILD.

There were two deaths during the study period. One person with isolated ANCA‐ILD died 8 years after initial diagnosis following an episode of *Pneumocystis jirovecii* pneumonia in the setting of immunosuppression with glucocorticoids. A second death occurred in a person with MPA‐ILD who was initially noted to have a probable UIP pattern during a lung cancer screening trial. They progressed to develop MPA features with rising antibody titres, were treated with glucocorticoids and ultimately died 3 years after diagnosis from a slow clinical decline in the setting of progressive pulmonary fibrosis and *Mycobacterium avium* complex infection.

## 3 Discussion

The most striking finding from this series was the association between a UIP radiological pattern and concurrent or subsequent diagnosis of MPA‐ILD. Most people with isolated ANCA‐ILD presenting with a UIP radiological pattern went on to develop MPA‐ILD (80%) and this was also the most common pattern seen in those with MPA‐ILD. Those with MPA‐ILD had a significantly higher median number of admissions to hospital during follow up (3 [IQR 3–9] in MPA‐ILD vs. 2 [IQR 1–3] in isolated ANCA‐ILD), reflecting MPA's multisystem involvement and associated morbidity. We found that people with MPA‐ILD had a lower 6MWT distance achieved at time of diagnosis. They also had a higher baseline mean ESR suggesting a higher degree of systemic inflammation, although this did not reach significance. High ESR has been shown to be associated with ANCA seroconversion among people with ILD and is an independent risk factor for mortality in isolated ANCA‐ILD or MPA [13, 14].

In our series, we found a similar mean lag of 16.6 months between baseline and peak MPO‐antibody titres among those with eventual diagnosis of MPA and those with isolated ANCA‐ILD. However, high baseline and peak MPO‐antibody titres seem to be associated with eventual diagnosis of MPA‐ILD. This finding strengthens the argument for routine antibody monitoring among this cohort, particularly in those with high baseline titres or evolving clinical features [13, 14]. Recent reporting demonstrates progression rates from isolated ANCA‐ILD to AAV of 4.2% per year [2].

We identified all ILD patients with MPO‐antibody positivity seen in the Respiratory clinic at our tertiary centre, allowing for detailed follow‐up over a mean of over 4 years. This allowed description of the natural course of this condition and factors associated with progression of MPA. All patients had MDT consensus diagnoses of ILD at baseline, consistent with current guidelines [10]. Our case series has several limitations. Firstly, current guidelines do not suggest ANCA testing for ILD workup in the absence of features of vasculitis. Our centre's practice is to include ANCA testing, but heterogeneity in physicians' practice may have led to cases of isolated ANCA‐ILD being missed raising the possibility of selection bias. Additionally, as a retrospective case series, we are unable to infer causality. Despite the rarity of MPO‐antibody positivity in ILD, our sample size was small and lacked statistical power to investigate risk factors for developing MPA‐ILD.

Our results are consistent with previous reporting in demonstrating that a UIP radiological pattern is associated with the development of MPA‐ILD [2, 7, 13]. They are also consistent with recent publications suggesting that ANCA testing should be routine as part of the initial ILD workup. This would identify patients with isolated ANCA‐ILD who are at a higher risk of progression to MPA‐ILD [7, 13, 15].

Our findings also highlight the importance of ongoing surveillance of MPO‐antibody titres and clinical features of vasculitis on follow‐up, as MPO‐antibody positivity and features of MPA‐ILD may manifest many years later. This aligns with current literature emphasising the need for long‐term monitoring of these patients [7, 13, 14].

Management of people with isolated ANCA‐ILD remains challenging, and it is unclear if they should be managed as MPA‐ILD or idiopathic pulmonary fibrosis [13]. This treatment heterogeneity for people with isolated ANCA‐ILD was observed in our case series, where 44% received immunosuppression compared to 100% of those with MPA‐ILD [6, 13]. It is also uncertain if those with isolated ANCA‐ILD have a more favourable prognosis than those with MPA‐ILD, likely due to the heterogeneity of this group among studies given ANCA testing is not routinely recommended as part of the ILD workup [6, 13]. Those with isolated ANCA‐ILD saw a greater loss in 6MWT distance and total lung capacity per year than those with MPA‐ILD, acknowledging the small sample size with multiple tests available for comparison. This may represent appropriate immunosuppression of those with MPA‐ILD and highlights difficulty in the treatment of those with isolated ANCA‐ILD.

MPA‐ILD has been shown to be associated with increased mortality compared with isolated ANCA‐ILD [3]. In our series, there was one death in each of these groups over the study period. It remains unclear if steroids worsen outcomes in those with isolated ANCA‐ILD and immunosuppression can carry significant risk [6].

Half of those that developed MPA were initially diagnosed with ILD incidentally in the absence of systemic vasculitis symptoms, with these features developing on interval follow‐up. This may be increasingly common due to increased case ascertainment from cross‐sectional coronary imaging and/or lung cancer screening.

In summary, our findings support the recommendation that all newly diagnosed ILD cases be referred to a tertiary ILD specialist centre for MDT review. This enables MDT discussion for diagnostic and treatment consensus and ongoing joint management between Respiratory, Immunology and Rheumatology physicians, an approach consistent with current best practices in ILD management [10, 13].

## Author Contributions

Michael Hoskins was involved in the conceptualisation, methodology, data collection and interpretation, writing, editing and final approval of the manuscript. Grace Thompson was involved in the supervision, conceptualisation, methodology, reviewing, editing and final approval of the manuscript. Vidya Navaratnam was involved in the supervision, conceptualisation, methodology, reviewing, editing and final approval of the manuscript.

## Ethics Statement

This study was approved by Sir Charles Gairdner Osborne Park Health Care Group Human Research Ethics Committee, reference number 42518.

## Conflicts of Interest

The authors declare no conflicts of interest.

## Data Availability

The data that support the findings of this study are available on request from the corresponding author. The data are not publicly available due to privacy or ethical restrictions.
